# Association between prenatal maternal cigarette smoking and early childhood caries. A systematic review

**DOI:** 10.4317/jced.54064

**Published:** 2017-09-01

**Authors:** Sergio-Varela Kellesarian, Vanessa-Ros Malignaggi, Paula de Freitas, Hameeda-Bashir Ahmed, Fawad Javed

**Affiliations:** 1 Department of General Dentistry, Eastman Institute for Oral Health, University of Rochester, New York, USA; 2 Department of General Dentistry, Universidad Santa Maria, Caracas, Venezuela; 3 Private Dental Practice, Doha, Qatar

## Abstract

**Background:**

The aim of the present study was to assess the relationship between prenatal maternal cigarette smoking (PMCS) and early childhood caries (ECC) through a systematic review of currently available scientific evidence.

**Material and Methods:**

To address the focused question: “Is there an association between PMCS and ECC?” an electronic literature search without time or language restrictions was conducted till May 2017 in indexed databases using various key words including dental caries, pregnancy, smoking, tobacco products and child. Letters to the editor, commentaries, reviews, case reports and case series and studies in which, ECC was investigated without clinical dental examination, were excluded.

**Results:**

Eight observational cross-sectional studies were included. The number of participants ranged between 1102 and 76920 children with age ranging between 24 months and 72 months. Seven studies reported a positive association between PMCS and ECC. One study reported that children whose mother smoked at least five cigarettes/day during pregnancy presented a higher caries severity level compared with to those whose mothers did not smoke. One study showed no association between ECC and PMCS.

**Conclusions:**

The association between PMCS and ECC remains debatable. Further well-designed longitudinal studies are needed in this regard.

** Key words:**Cigarette, early childhood caries, pregnancy, risk factors, smoking.

## Introduction

Cigarette smoking (CS) is a classical risk-factor of lethal diseases such as lung cancer and cardiovascular anomalies (1). From a dental perspective, CS jeopardizes periodontal and oral mucosa health by increasing the risk of periodontitis and oral precancer and cancer, respectively (2). Some studies (3,4) have also shown that the risks of dental caries are higher in cigarette-smokers compared with non-smokers. One explanation in this regard is that CS reduces the salivary flow rate, which increases the accumulation of the oral biofilm (that contains cariogenic microbes), on teeth surfaces thereby increasing the risk of dental caries (5,6).

Prenatal maternal CS (PMCS) has been associated with complications including preterm birth, low birth weight, birth defects, and sudden infant death syndrome (7-11). Post-natal conditions, including increased childhood bone fractures, mental disorders, and respiratory disorders have also been associated with PMCS (12-15). Interestingly, a limited number of studies (16-23) have explored the association between early childhood caries (ECC) and PMCS. Iida *et al.* (17) used data from the United States National Health and Nutrition Examination Survey (NHANES) to assess the association of maternal smoking, breastfeeding and other factors with ECC. The findings suggested that children of females that smoked during pregnancy are at a greater risk of having decayed and filled surfaces on deciduous teeth surfaces (17) compared with children of non-smoking females. Similar results have been reported in other epidemiological studies (18,20-22). However, conflicting results have been also reported. Shulman *et al.* (16) did not find an association between ECC and maternal risk factors, including PMCS.

With this background, the aim of the present study was to assess the relationship between PMCS and ECC through a systematic review of currently available scientific evidence.

## Material and Methods

Based on the Preferred Reporting Items for Systematic Reviews and Meta-Analyses guidelines, a specific question was constructed according to the PECO (Participants, Exposure, Comparative, Outcomes) principle (24). The addressed focused question was: “Is there an association between PMCS and ECC?” 

-Eligibility criteria 

The eligibility criteria were as follows: (a) original observational studies (cohort, cross-sectional or case-control) that evaluated the association between PMCS and ECC; (b) studies with a comparative group (children not exposed to PMCS). Letters to the editor, commentaries, reviews, case reports and case series, studies where ECC was diagnosed without dental examination and studies related to the prevalence of caries in adolescents were excluded.

-Literature search protocol and data extraction

The Cochrane Register of Systematic Reviews and the International Database of Prospectively Registered Systematic Reviews in Health and Social Care and were searched in May 2017, and presented no existing reviews assessing the association between PMCS and ECC. In order to identify studies relevant to the focused question, a systematic and structured literature search without language restrictions was conducted by two authors (SVK and FJ) using PubMed (National Library of Medicine, Bethesda), Scopus, EMBASE, and Web of Knowledge databases. The databases were searched up to and including May 2017 using different combinations of the following Medical Subject Headings (MeSH) terms: (1) dental caries; (2) pregnancy; (3) smoking; (4) tobacco products; and (5) child. Other related non-MeSH terms were used in the search strategy to detect articles discussing the association between PMCS and ECC. These included: (6) teeth decay; (7) pregnant and (8) maternal and (9) cigarette. These keywords were used with Boolean operators (OR, AND) to combine the key words mentioned above: (a) 1 or 6, and 2 or 7 or 8, and 3 or 4 or 9; (b) 1 or 6, and 2 or 7 or 8, and 3 or 4 or 9, and 5.

To reduce the potential for reviewer bias, titles and abstracts of studies identified using the above-described protocol were independently screened by 2 authors (SVK and FJ) and checked for agreement. Full-texts of studies judged by title and abstract to be relevant were read and independently evaluated for the stated eligibility criteria. After initial electronic search, references of the identified studies were hand-searched to identify further potentially relevant studies. Any disagreements in the study selection were resolved via discussion and consensus between the authors (SVK and FJ). Cohen’s kappa value (25) was used to determine the inter-reviewer reliability. (Kappa score = 0.90). Data was extracted using standardized evaluation forms. Authors of the studies included were contacted via electronic mail in case data was missing or additional information regarding their studies was required.

-Quality assessment 

The Newcastle – Ottawa Scale (NOS) (26) was used to grade the methodological quality of studies included in the present review. In summary the NOS scale uses a systematic approach based on 3 specific criteria: Selection (S), Comparability (C) and Exposure (E), which are subdivided in 9 criteria: S1) Adequate case definition; S2) Representativeness of the cases; S3) Selection of control; S4) Definition of control; C1) Comparability of cases; C2) Controls on the basis of the analysis; E1) Ascertainment of exposure; E2) Same method of ascertainment for cases and controls; E3) Non-response rate. Each criterion was given a response of either “Yes”, “No”, or ‘‘cannot tell,’’. Each study could have a maximum score of 9. Quality assessment of studies included was conducted independently by two authors (SVK and FJ) using the above-described tool. Qualitative analyses were checked for disagreement via discussion among the authors. (Kappa score = 0.82).

## Results

-Study selection 

**Figure 1:**
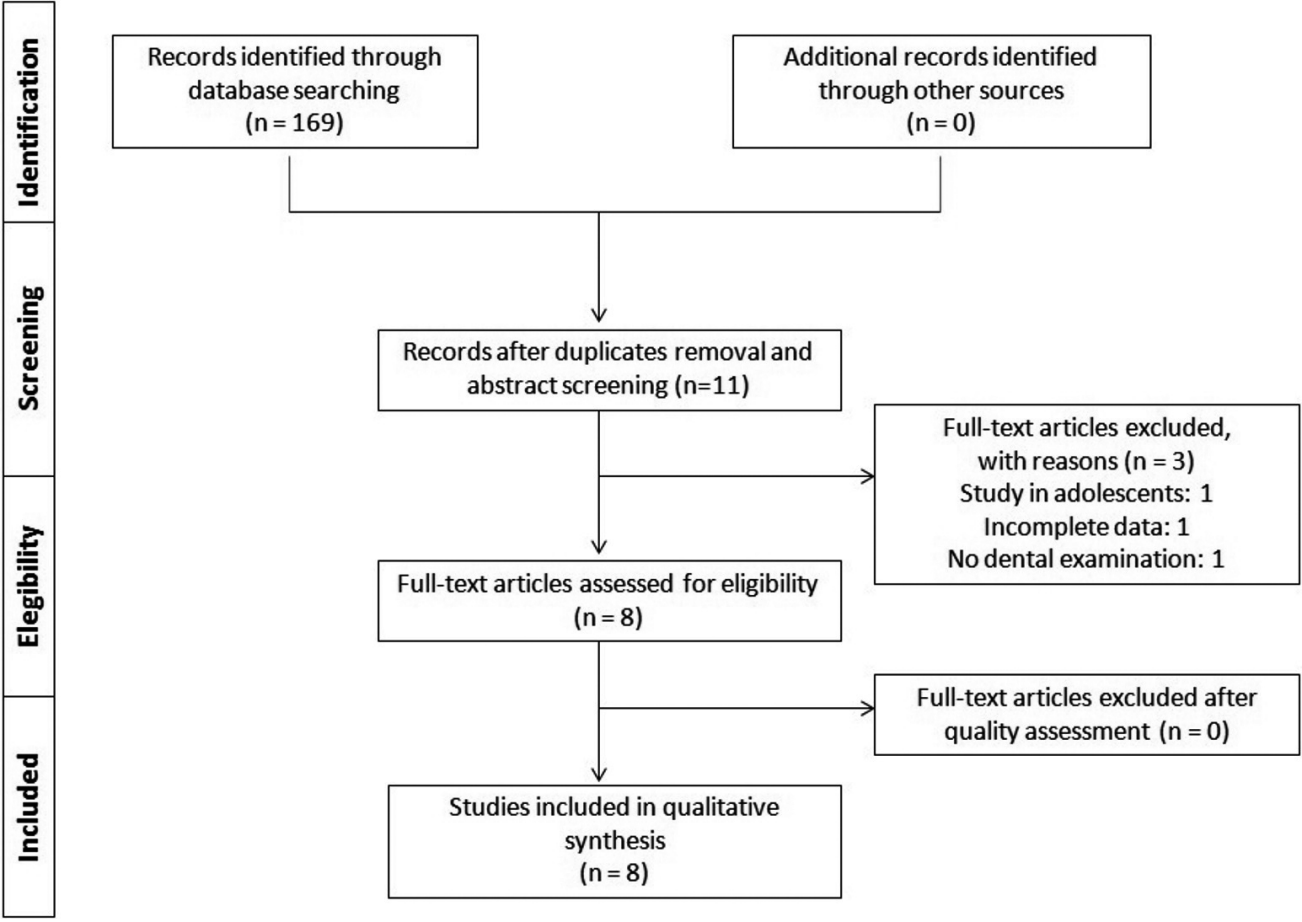
Article selection flow chart for the systematic review according to PRISMA guidelines.

One hundred and sixty-nine potential articles were initially identified. After title and abstract screening 158 publications were excluded (duplicates or did not answer the focused question). In the second step, after full-text evaluation 3 more articles were excluded (Appendix A). A total of 8 studies (16-23) were included for qualitative analysis. Figure 1 summarizes the study selection process.

-General characteristics

All studies (16-23) presented an observational cross-sectional design. These primary studies (16-23) were conducted between 2005 and 2017 in the following countries: Italy, Japan, Scotland and United States. In all studies (16-23), the number of study participants ranged between 1102 and 76920 children with age ranging between 24 months and 72 months. In 6 studies (16-18,20-22), the data was obtained from national cross-sectional studies, out of which, 2 studies (16,17) used data from the US NHANES ; whereas, 4 studies (18,20-22) conducted in Japan used data from the Fukuoka Child Health Study, the Kobe Offspring Study or the Kyushu Okinawa Child Health Study. Bernabe *et al.* (23) used data from a 4 years longitudinal caries risk assessment study conducted in Scotland. Majorana *et al.* (19) conducted a retrospective study among Italian children to address the association between dental caries in children and maternal smoking habits during pregnancy.

-Pre-natal Maternal Cigarette Smoking

In all studies (16-23), PMCS was self-reported. None of the studies (16-23) reported the number of cigarettes per day, duration or frequency of smoking during pregnancy. Majorana *et al.* (19) defined smoking during pregnancy as positive when the mother reported smoking more than 5 cigarettes per day. One study (18), classified the maternal smoking during pregnancy into 3 categories: none, stopped at some time during pregnancy, and smoked throughout pregnancy. Whereas, Tanaka *et al.* (22) classified maternal smoking status in 4 categories: none, first trimester only, second and/or third trimesters but not throughout pregnancy and smoked throughout pregnancy. Five studies (18-22) assessed postnatal exposure to household smoking in addition to PMCS.

-ECC assessment

In all studies (16-23) the assessment of ECC was made by oral examination (visual and/or tactile). None of the studies (16-23) used radiographs in the diagnosis of ECC. Three studies (16,17,22) used the decayed or filled primary tooth surface (DFS) index. Hence, in these studies (16,17,22) ECC refers to the presence of any DFS on any primary tooth. In 3 studies (18,20,21), children were classified as having ECC if one or more of the primary teeth were decayed, missing or filled (DMF). Bernabe *et al.* (23) calculated the number of decayed, missing, and filled tooth surfaces (DMFS index) as outcome measure. In one study (19), the severity of caries lesions was classified using the International Caries Detection and Assessment System (ICDAS): low (ICDAS 1-3), moderate (ICDAS 4) and high (ICDAS 5 and 6) (T Table 1).

**Table 1:**
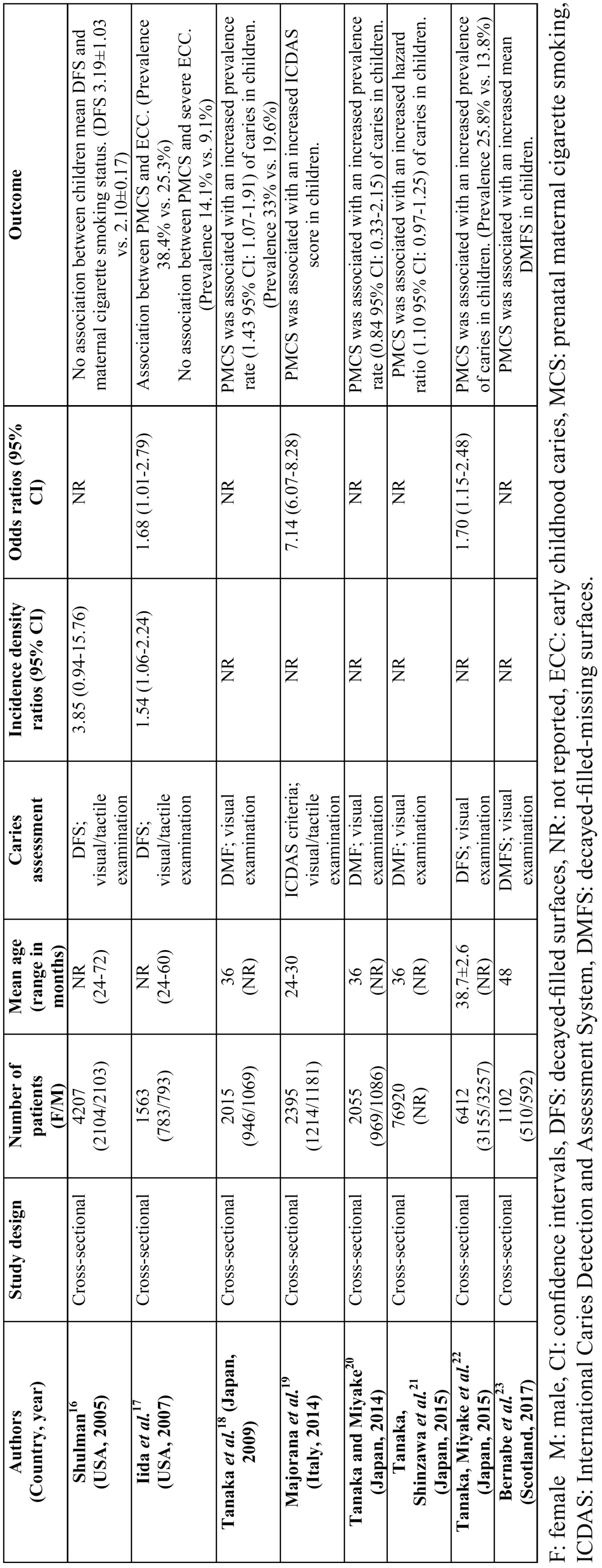
Characteristics of the studies included.

-Confounding factors

In all studies (16-23) data was adjusted for confounding factors including maternal co-variants (such as, age at child’s birth, education and/or socioeconomic position) and child factors (including breastfeeding duration, sweet dietary habits and/or oral hygiene habits).

-Main outcomes

Seven studies (17-23) reported a positive association between PMCS and ECC. Four studies (18,20-22) reported that PMCS was associated with an increased prevalence rate of dental caries in children. Majorana *et al.* (19) reported that children whose mother smoked five or more cigarettes/day during pregnancy presented a higher caries severity level compared with to those whose mothers did not smoke (OR: 7.14, 95% CI: 6.07-8.28). Iida *et al.* (17) reported that PMCS was an independent risk factor for ECC. Bernabe *et al.* (23) reported that PMCS was associated with an increased mean DMFS in children; whereas, Shulman *et al.* (16) showed no association between children mean DFS and PMCS. It is pertinent to mention that the significant heterogeneity among all the studies (16-23) did not allow pooling of the results and statistical analysis.

-Quality Assessment of included studies

The total quality score among the included studies (16-23) ranged between 8 and 9. The most common limitation among all studies (16-23) was the inaccurate measurement of exposure (PMCS) and outcome (ECC). On average, the quality of included studies (16-23) was good, but the aforementioned shortcomings limit the application of these study findings. Quality assessment of the individual papers is summarized in  Table 2.

**Table 2:**
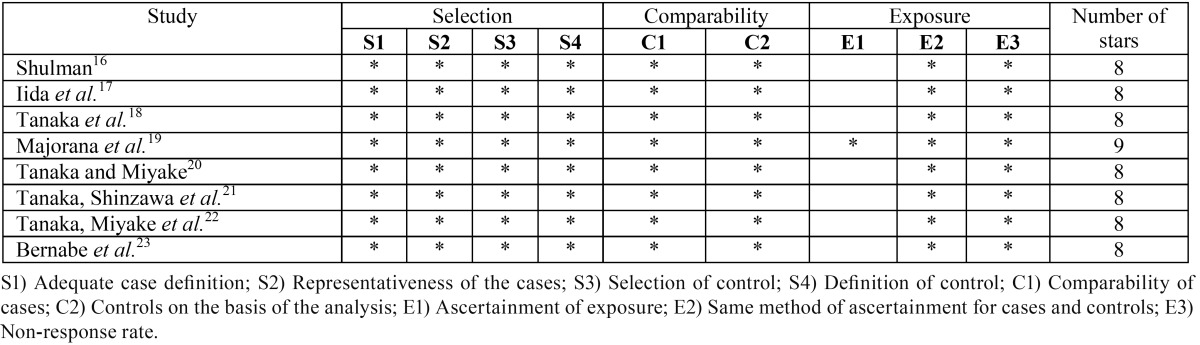
Assessment of study quality with Newcastle-Ottawa Scale.

## Discussion

According to the American Academy of Pediatric Dentistry, ECC is defined as the presence of 1 or more decayed (noncavitated or cavitated lesions), missing (due to caries), or filled tooth surfaces in any primary tooth in a child 71 months old or younger (27); whereas, in children younger than 3 years of age, any sign of smooth-surface caries is indicative of severe ECC. From ages 3 through 5, 1 or more cavitated, missing (due to caries), or filled smooth surfaces in primary maxillary anterior teeth or a decayed, missing, or filled score of ≥4 (age 3), ≥5 (age 4), or ≥6 (age 5) surfaces constitutes severe ECC (27). From the literature reviewed, nearly 88% of the studies (17-23) showed a positive association between PMCS and ECC. This suggests that PMCS is a risk-factor of ECC. However, a number of factors may have biased the results reported in the studies (16-23) included in the present systematic review. Firstly, it is well known that oral inflammatory conditions are worst in individuals with a higher daily frequency of CS as compared to individuals smoking a significantly lower number of cigarettes daily and non-smokers (28). It is pertinent to mention that only in the study by Majorana *et al.* (19) the minimum daily frequency of PMCS was reported (>5 cigarettes/day). From this study (19) it seems that women who smoked >5 cigarettes/day exposed children to increased risk of ECC as compared to non-smoking pregnant women. None of the remaining studies addressed this issue (16-18,20-23). It is therefore difficult to establish a threshold for daily CS during pregnancy that may expose the future child to ECC. It is hypothesized from the results of Majorana *et al.* (19) that women smoking at least 10 cigarettes/day would significantly increase the risk of ECC in the future offspring as compared to females smoking less than 5 cigarettes during pregnancy. Therefore, additional well-designed power adjusted studies are needed to test this hypothesis.

Another factor that might have influenced the results of the included studies (16-23) is the smoking habit duration. It is noteworthy that none of the studies that fulfilled our eligibility criteria assessed the duration of smoking. It is speculated that the offspring of women with a longer history of smoking are more prone to ECC as compared to females with a shorter duration of CS. Moreover, 7 out of 8 studies (16-21,23) did not take in account the stages of pregnancy (first, second or third trimesters) during which the mothers smoked. Tanaka *et al.* (22) reported that PMCS during the first trimester resulted in highest risk of ECC. By no means have the authors of the present review suggested that PMCS is safe during any stage of pregnancy, but still remains to be elucidated which trimester of pregnancy is more susceptible for ECC.

The authors of the present systematic review propose that besides offering treatment to children with ECC, it is also imperative for oral healthcare providers to educate their parents/guardians, about the detrimental effects of smoking on health particularly during pregnancy. It is recommended that routine community health awareness programs should be conducted to educate the public about the deleterious effect of habits such as smoking on health and also to emphasize encourage the public to visit their dental and general healthcare providers for routine check-ups. This may help improve the quality of life of not only individuals but also their children.

## Conclusions

There is insufficient evidence to justify that an association exists between PMCS and ECC. Therefore, further well-designed longitudinal studies with a statistically-justified sample size are needed in this regard.
